# Air Pressure an Available Force in Retentive Dressing for Fractures of the Upper Dental Arcade*Presented before the Wisconsin State Medical Society at its annual meeting, May 4th, 1892.

**Published:** 1892-12

**Authors:** J. R. Barrett

**Affiliations:** Neenah, Wis.; Ex-President Wisconsin State Medical Society; Ex-President Fox River Valley Medical Society


### AIR PRESSURE AN AVAILABLE FORCE IN RETENTIVE DRESSING FOR FRACTURE OF THE UPPER DENTAL ARCADE.*

BY J. R. BARRETT, M. D., NEENAH, WIS.
Ex-President Wisconsin State Medical Society ; Ex-President Fox River Valley Medical Society.

Systematic writers on fractures give but little space to such accidents befalling the upper jaw.

Bearing so little relation to the importance of such injuries, it must be a confession that but little needs to be done, or on the other hand, that but little can be done, except in those violent disjunctions that sometimes leave the superior maxil-

*Presented before the Wisconsin State Medical Society at its annual meeting, May 4th, 1892.

laries widely separated from each other or form other articulating bones.

This graver class of injuries will not here be considered, nor the milder ones involving simply fracture without separation.

The commonest injuries to the upper jaw coming to the attention of the surgeon are those involving the integrity of the dental arcade, and these may be limited to a loosening of one or more teeth in their sockets, or include a fracture of the alveolar process with displacement, or a still more extensive one into the body or palate process, occurring in the molar region, and unattended by great displacement; they present few difficulties in the way of fair coaptation and fixation; but in the more anterior region of the jaw, especially that portion holding the incisor teeth, exact coaptation is rendered more imperative for cosmetic effect, while fixation is more difficult, owing to the imperfect and varying support of the inferior dental arcade. In the former region, in the majority of cases, there is but the slight muscular action of the buccinator, hardly worth considering, to throw the fragment out of place, and at the worst, that of the lower head of the external pterygoid, having its insertion in the lower jaw, and consequently relaxing under the very manœuvre resorted to immobilize the fracture—namely, the closure of the lower jaw, and constant pressure of the lower against the upper teeth, either mediately or immediately. Again, in this region there is not that temptation to exercise movements of the lower jaw under retentive dressings which is especially strong with young patients; for only the violent action of the depresssors could relieve the fragment from pressure, whereas lateral and anterior-posterior movements, while not relieving the teeth from contact and consequent support, could not fail to be too painful to tempt to frequent repetition.

In the incisor region these conditions are changed. Unless the fracture reaches up into the body of the bone, there is no muscular attachment to affect the fragment, but it can be wholly released from support by backward movements of the lower jaw, which no form of bandage can wholly prevent, and which the perversity of many patients, both young and old, will surely make the most of, especially when pain from pressure is at all severe.

These difficulties in the way of retentive dressings are real, and not in the least exaggerated, as all who have treated fractures of the jaws will recognize. Indeed, they seem to be the principal difficulties, taxing the ingenuity of the surgeon more than these presented by involuntary muscular contraction, which can be exactly anticipated and more or less exactly obviated.

What treatment for this limited class of cases have surgeons proposed? Hamilton says that "when the harmonies of the upper maxillary bones are but slightly disturbed, nothing but a retentive treatment is necessary." Later he says, "in some instances, when fragments are displaced, carrying with them several teeth, while others in the same row remain firm, it will be sufficient to close the mouth and apply a bandage as for fracture of the inferior maxilla; in others, the teeth and their alveola ought to be fastened with silk, or gold, or silver thread. Gold, silver, gutta-percha or vulcanite clasps may be applied to the teeth and jaw."

This is the best statement of alternatives that I have seen; but the first mistrial will be wholly insufficient, as I have pointed out; the second is less practicable than it seems, where it is desirable to preserve the exact contour of the dental arch, as in women; besides it runs the risk of entirely severing the loosened fragment of bone where its attachment is slight, thus defeating the very object of surgical intervention.

He does not elaborate the method by metal or vulcanite clasps, but evidently it involves external, additional support, like the retention of the lower jaw closed.

Agnew makes still less of the minor fracture. "When a portion of the alveolus is broken away," he says, "either from its inner or outer wall * * no dressing is demanded." But he directs, when the process is broken into two or more fragments, that the pieces shall be pressed into place and held by the lowr jaw, which is to be brought up and secured by a bandage, the Barton or Gibson being particularly recommended. "It may happen," he says, "that the separated piece may die; if so, as soon as it is sufficiently loose, it should be removed with a pair of bone forceps."

This simplifies the duties and responsibilities of the surgeon exceedingly; but how shall the fair patient, more terrified by the risk of disfigurement than by the pain of her injury, look upon what may well seem to her such doubtful resources of the surgical art. It is but poor comfort to tell her that, if her teeth do not grow more or less in place, the dentist can by-and-by fili the gap with artificial ones. It needs not that the patient should be a woman to cherish the natural teeth as far preferable to the porcelain kind; and it does seem to me that the method proposed does not give him that relative guaranty of perfect repair to which he is entitled. The pressure of the lower teeth against the upper fragment does not always retain it in position when once placed there. If the displacement is backward, as it almost always is, the retentive dressing should carry the lower jaw forward, as well as upward, to hold the opposing incisors in contact. But the Barton and all similar bandages carry the jaws upward and backward, a function which Agnew later on recognizes and avails himself of in the dressing he proposes for fracture of the inferior maxilla.

Neither these nor other writers neglect to mention the Gumming interdental splint and its various modifications, or other more or less complicated and difficult dressings, in the more serious fractures; but all rely too much upon the well known reparative power of the upper maxilla to remedy the damage with but little help, when the damage is confined wholly or mainly to the alveolus. They would seem to be satisfied with union of the fracture, which is easily attained, even if the position is not entirely perfect.

That we can almost certainly secure a perfect result, not only functionally but cosmetically, I think will be shown by the following outline history of a case:

Miss O., age 12, was accidentally struck in the mouth by a croquet mallet, the blow fracturing the upper incisor alveoli, producing backward and downward displacement of the central teeth to the distance of nearly one-fourth of an inch. Firm and steady pressure, continued for some minutes, brought the piece nearly but not quite into position; but soon separation returned when pressure was removed.

The treatment employed was the customary one of bringing up the lower jaw and bandaging in firm position. I was sorry to find the next morning that the fragment was not in place; and then my patient told me that as there was some pain she would often draw the lower jaw back for the comfort it would afford. This could be done to the extent of one-fourth of an inch, and in spite of the bandage, the jaw could be depressed quite as much. Foreseeing that a perfect result could not be got in this manner, I had an impression of the fractured jaw taken, and a vulcanite splint made to fit. Dr. Tinker, a dentist of our city, did this with great skill; and when it was applied, the suction or atmospheric pressure was sufficient to hold it in position, as a plate of artificial teeth is held. It is likely that this force could have been quite sufficient from the first, but it seemed best to give the additional security of the lower jaw bandaged up for a few days, especially as coaptation was not at once exact, and continued firm pressure seemed necessary to complete it.

At the end of three days the splint was carefully detached, and the contour of the dental arch found to be exactly restored. After this the bandage was dispensed with entirely, and at the end of four weeks union in position was perfect.

It is not unlikely that others have, in the treatment of such fractures of the upper jaw, availed themselves of the retentive force of atmospheric pressure; but in such literature as has been accessible, I have not found any record of it. For this reason, my own case seemed deserving of a report; for, if not unique, it may direct attention to a method which, to say the least, should be more generally recognized and employed.

Perhaps the question may be raised as to the necessary size or superficial area of the palatal plate of such a splint. For limited practices this need not be great. The one I used did not exceed three-fourths of an inch in breadth and was consequently horse-shoe shaped. Less might have sufficed for the pressure over two square inches where the fit is exact, and the air underneath well exhausted, is a strong retentive force, as you will readily perceive. There might, however, be the necessity for embracing the whole hard palate; though it is doubtful if the retentive force would be proportionate to the increased area, owing principally to the increased difficulty of securing an exact fit.

In the usual form of fracture with inward displacement, the dental groove in the splint is not a theoretical necessity; but for precautionary reasons it would be better to have it; if not for the purpose of greater security against accident, at least to provide against the contingency of an ill fit when it could be made to serve the purpose of an ordinary interdental splint. It seems probable, however, in cases where the fit is not primarily good, that nature may, after a day or two, come to the aid of the dentist, and then dam the leaky places in the palate process with swollen tissues.

